# A Robust Phylogenomic Time Tree for Biotechnologically and Medically Important Fungi in the Genera *Aspergillus* and *Penicillium*

**DOI:** 10.1128/mBio.00925-19

**Published:** 2019-07-09

**Authors:** Jacob L. Steenwyk, Xing-Xing Shen, Abigail L. Lind, Gustavo H. Goldman, Antonis Rokas

**Affiliations:** a Department of Biological Sciences, Vanderbilt University, Nashville, Tennessee, USA; b Department of Biomedical Informatics, Vanderbilt University School of Medicine, Nashville, Tennessee, USA; c Gladstone Institute for Data Science and Biotechnology, San Francisco, California, USA; d Departamento de Ciências Farmacêuticas, Faculdade de Ciências Farmacêuticas de Ribeirão Prêto, Universidade de São Paulo, São Paulo, Brazil; University of Pittsburgh

**Keywords:** Ascomycota, Eurotiales, Eurotiomycetes, genomics, incongruence, International Code of Nomenclature, narrow *Aspergillus*, phylogenetics, phylogenomics, secondary metabolism

## Abstract

The filamentous fungal family Aspergillaceae contains >1,000 known species, mostly in the genera *Aspergillus* and *Penicillium*. Several species are used in the food, biotechnology, and drug industries (e.g., Aspergillus oryzae and Penicillium camemberti), while others are dangerous human and plant pathogens (e.g., Aspergillus fumigatus and Penicillium digitatum). To infer a robust phylogeny and pinpoint poorly resolved branches and their likely underlying contributors, we used 81 genomes spanning the diversity of *Aspergillus* and *Penicillium* to construct a 1,668-gene data matrix. Phylogenies of the nucleotide and amino acid versions of this full data matrix as well as of several additional data matrices were generated using three different maximum likelihood schemes (i.e., gene-partitioned, unpartitioned, and coalescence) and using both site-homogenous and site-heterogeneous models (total of 64 species-level phylogenies). Examination of the topological agreement among these phylogenies and measures of internode certainty identified 11/78 (14.1%) bipartitions that were incongruent and pinpointed the likely underlying contributing factors, which included incomplete lineage sorting, hidden paralogy, hybridization or introgression, and reconstruction artifacts associated with poor taxon sampling. Relaxed molecular clock analyses suggest that Aspergillaceae likely originated in the lower Cretaceous and that the *Aspergillus* and *Penicillium* genera originated in the upper Cretaceous. Our results shed light on the ongoing debate on *Aspergillus* systematics and taxonomy and provide a robust evolutionary and temporal framework for comparative genomic analyses in Aspergillaceae. More broadly, our approach provides a general template for phylogenomic identification of resolved and contentious branches in densely genome-sequenced lineages across the tree of life.

## Abstract (precis)

Understanding the evolution of traits across technologically and medically significant fungi requires a robust phylogeny. Even though species in the *Aspergillus* and *Penicillium* genera (family Aspergillaceae, class Eurotiomycetes) are some of the most significant technologically and medically relevant fungi, we still lack a genome-scale phylogeny of the lineage or knowledge of the parts of the phylogeny that exhibit conflict among analyses. Here, we used a phylogenomic approach to infer evolutionary relationships among 81 genomes that span the diversity of *Aspergillus* and *Penicillium* species, to identify conflicts in the phylogeny, and to determine the likely underlying factors of the observed conflicts. Using a data matrix comprised of 1,668 genes, we found that while most branches of the phylogeny of the Aspergillaceae are robustly supported and recovered irrespective of method of analysis, a few exhibit various degrees of conflict among our analyses. Further examination of the observed conflict revealed that it largely stems from incomplete lineage sorting and hybridization or introgression. Our analyses provide a robust and comprehensive evolutionary genomic roadmap for this important lineage, which will facilitate the examination of the diverse technologically and medically relevant traits of these fungi in an evolutionary context.

## INTRODUCTION

The vast majority of the 1,062 described species from the family Aspergillaceae (phylum Ascomycota, class Eurotiomycetes, order Eurotiales) (1) belong to the genera *Aspergillus* (42.5%; 451/1,062) and *Penicillium* (51.6%; 549/1,062) (2, 3). Fungi from Aspergillaceae exhibit diverse ecologies: for example, Penicillium verrucosum is widespread in cold climates but has yet to be isolated in the tropics (4), whereas Aspergillus nidulans is able to grow at a wide range of temperatures but favors warmer ones (5). Several representative species in the family are exploited by humans, while a number of others are harmful to humans or their activities (6). Examples of useful-to-humans organisms among *Aspergillus* species include Aspergillus oryzae, which is used in the production of traditional Japanese foods, including soy sauce, sake, and vinegar (7, 8), as well as of amylases and proteases (9), and Aspergillus terreus, which produces mevinolin (lovastatin), the potent cholesterol-lowering drug (10). Examples of useful-to-humans *Penicillium* species include Penicillium camemberti and Penicillium roqueforti, which contribute to cheese production (11, 12), and Penicillium citrinum, which produces the cholesterol-lowering drug mevastatin, the world’s first statin (13). In contrast, examples of harmful-to-humans organisms include the pathogen, allergen, and mycotoxin-producing species Aspergillus fumigatus and Aspergillus flavus (14, 15) and the postharvest pathogens of citrus fruits, stored grains, and other cereal crops Penicillium expansum, Penicillium digitatum, and Penicillium italicum (16–18).

Much of the ubiquity, ecological diversity, and wide impact on human affairs that Aspergillaceae exhibit is reflected in their phenotypic diversity, including their extremotolerance (e.g., ability to withstand osmotic stress and wide temperature range) (19–22) and ability to grow on various carbon sources (21, 23). Fungi from Aspergillaceae are also well known for their ability to produce a remarkable diversity of secondary metabolites, small molecules that function as toxins, signaling molecules, and pigments (24–29). Secondary metabolites likely play key roles in fungal ecology (30–32), but these small molecules often have biological activities that are either harmful or beneficial to human welfare. For example, the A. fumigatus-produced secondary metabolite gliotoxin is a potent virulence factor in cases of systemic mycosis in vertebrates (33), and the A. flavus-produced secondary metabolite aflatoxin is among the most toxic and carcinogenic naturally occurring compounds (24, 34). In contrast, other secondary metabolites are mainstay antibiotics and pharmaceuticals: for example, the Penicillium chrysogenum-produced penicillin is among the world’s most widely used antibiotics (35–37) and the *P. citrinum*-produced cholesterol-lowering statins are consistently among the world’s blockbuster drugs (13).

Species from Aspergillaceae have also served as model systems to understand fungal sexual and asexual development (38–40). From a biotechnological perspective, understanding of the genes and conditions required for sexual reproduction has been key for strain improvement: for example, taking advantage of knowledge of mating-type genes enabled the design of sexual crosses between P. chrysogenum strains that generated offspring with novel phenotypic combinations relevant to penicillin production (38). From a medical perspective, sexual reproduction contributes to the diversification of pathogens and may contribute to the spread of antifungal resistance (39): for example, evidence suggests that sexual reproduction in A. fumigatus has contributed to the diversification of drug-resistant isolates in Europe and may contribute to the spread of resistance (41). Last, from an evolutionary perspective, understanding the evolution of gene regulatory networks governing development and the formation of asexual spores, whose inhalation and germination are the major route to human infections (42), can aid in understanding the evolution of fungal development and pathogenicity. For example, it was recently shown that the regulatory cascade associated with asexual sporulation is functionally conserved across the genus *Aspergillus* but that the gene regulatory network downstream of *wetA*, the master regulator of spore development, has functionally diverged, shedding light into the similarities and differences of asexual spore evolution across the genus (40).

Understanding the evolution of the diverse ecological lifestyles exhibited by Aspergillaceae members as well as the family’s morphological and chemical diversity requires a robust phylogenetic framework. To date, most molecular phylogenies of the family Aspergillaceae are derived from single or few genes and have yielded conflicting results. For example, it is debated whether the genus *Aspergillus* is monophyletic or if it includes species from other genera such as *Penicillium* (43, 44). Furthermore, studies using genome-scale amounts of data, which could have the power to resolve evolutionary relationships and identify underlying causes of conflict (45, 46), have so far tended to use a small subset of fungi from either *Aspergillus* or *Penicillium* (23, 47, 48). Additionally, these genome-scale studies do not typically examine the robustness of the produced phylogeny; rather, based on the high clade support values (e.g., bootstrap values) obtained, these studies infer that the topology obtained is highly accurate (23, 47–49).

In recent years, several phylogenomic analyses have shown that high clade support values can be misleading (45, 50, 51); that incongruence, the presence of topological conflict between different data sets or analyses, is widespread (45, 52–54); and that certain branches of the tree of life can be very challenging to resolve, even with genome-scale amounts of data (55–60). Comparison of the topologies inferred in previous phylogenomic studies in Aspergillaceae (23, 47–49) suggests the presence of incongruence (see Fig. S1 posted at figshare, https://doi.org/10.6084/m9.figshare.6465011). For example, some studies have reported section *Nidulantes* to be the sister group to section *Nigri* (23), whereas other studies have placed it as the sister group to *Ochraceorosei* (48) (see Fig. S1 posted at figshare, https://doi.org/10.6084/m9.figshare.6465011).

A robust phylogeny of Aspergillaceae is also key to establishing a robust taxonomic nomenclature for the family. In recent years, the taxonomy of *Aspergillus* and *Penicillium* has been a point of contention due to two key differences among inferred topologies based on analyses of a few genes (61, 62). The first key difference concerns the placement of the genus *Penicillium*. One set of analyses places the genus as a sister group to *Aspergillus* section *Nidulantes*, which would imply that *Penicillium* is a section within the genus *Aspergillus* (62), whereas a different set of analyses suggests that the genera *Penicillium* and *Aspergillus* are reciprocally monophyletic (61). The second key difference concerns whether sections *Nigri*, *Ochraceorosei*, *Flavi*, *Circumdati*, *Candidi*, and *Terrei*, which are collectively referred to as “narrow *Aspergillus*,” form a monophyletic group (62) or not (61). Both of these differences are based on analyses of a few genes (4 loci [62] and 9 loci [61]), and the resulting phylogenies typically exhibit low support values for deep internodes, including for the ones relevant to this debate.

To shed light on relationships among these fungi, we employed a genome-scale approach to infer the evolutionary history among *Aspergillus*, *Penicillium*, and other fungal genera from the family Aspergillaceae. More specifically, we used the genome sequences of 81 fungi from Aspergillaceae spanning 5 genera, 25 sections within *Aspergillus* and *Penicillium*, and 12 outgroup fungi to construct nucleotide (NT) and amino acid (AA) versions of a data matrix comprised of 1,668 orthologous genes. Using three different maximum likelihood schemes (i.e., gene-partitioned, unpartitioned, and coalescence), we inferred phylogenies from the 1,668-gene data matrix as well as from five additional 834-gene data matrices derived from the top 50% of genes harboring strong phylogenetic signal according to five different criteria (alignment length, average bootstrap value, taxon completeness, treeness/relative composition variability, and number of variable sites). Using the same schemes, we also inferred phylogenies of the 1,668-gene data matrix using different alignment trimming methods as well as of a reduced 1,331-gene data matrix that was filtered for potential hidden paralogs. Comparisons of these phylogenies coupled with complementary measures of internode certainty (IC) (45, 63, 64) identified 11/78 (14.1%) incongruent bipartitions in the phylogeny of Aspergillaceae. These cases of incongruence can be grouped into three categories: (i) 2 shallow bipartitions with low levels of incongruence likely driven by incomplete lineage sorting, (ii) 2 shallow bipartitions with high levels of incongruence likely driven by hybridization or introgression (or very high levels of incomplete lineage sorting), and (iii) 7 deeper bipartitions with various levels of incongruence likely driven by reconstruction artifacts likely linked with poor taxon sampling. We also estimated divergence times across Aspergillaceae using relaxed molecular clock analyses. Our results suggest Aspergillaceae originated in the lower Cretaceous, 117.4 (95% credible interval [CI], 141.5 to 96.9) million years ago (mya), and that *Aspergillus* and *Penicillium* originated 81.7 mya (95% CI, 87.5 to 72.9) and 73.6 mya (95% CI, 84.8 to 60.7), respectively. We believe this phylogeny and time tree are highly informative with respect to the ongoing debate on *Aspergillus* systematics and taxonomy and provide a state-of-the-art platform for comparative genomic, ecological, and chemodiversity studies in this ecologically diverse and biotechnologically and medically significant family of filamentous fungi.

## RESULTS

### The examined genomes have nearly complete gene sets.

Assessment of individual gene set completeness showed that most of the 93 genomes (81 in the ingroup and 12 in the outgroup) used in our study contain nearly complete gene sets and that all 93 genomes are appropriate for phylogenomic analyses. Specifically, the average percentage of Benchmarking Universal Single-Copy Orthologs (BUSCO) single-copy genes from the Pezizomycotina database (65) present was 96.2% ± 2.6% (minimum, 81.1%; maximum, 98.9%) (see Fig. S2 posted at figshare, https://doi.org/10.6084/m9.figshare.6465011). Across the 93 genomes, only 3 (3.2%) genomes had <90% of the BUSCO genes present in single copy (Penicillium carneum, 88.6%; Penicillium verrucosum, 86.1%; and Histoplasma capsulatum, 81.1%).

### The generated data matrices exhibit very high taxon occupancy.

The NT and AA alignments of the 1,668-gene data matrix were comprised of 3,163,258 and 1,054,025 sites, respectively. The data matrix exhibited very high taxon occupancy (average gene taxon occupancy, 97.2% ± 0.1%; minimum, 52.7%; maximum, 100%; see Fig. S7a and b and File S2 at figshare, https://doi.org/10.6084/m9.figshare.6465011). Four hundred seventeen genes had 100% taxon occupancy, 1,176 genes had taxon occupancy in the 90% to 99.9% range, and only 75 genes had taxon occupancy lower than 90%. Assessment of the 1,668 genes for five criteria associated with strong phylogenetic signal (gene-wise alignment length, average bootstrap value, completeness, treeness/relative composition variability [RCV], and the number of variable sites) facilitated the construction of five subsampled matrices derived from 50% of the top-scoring genes (see Fig. S7 and File S2 posted at figshare, https://doi.org/10.6084/m9.figshare.6465011).

Examination of the gene content differences between the 5 NT subsampled data matrices as well as between the 5 AA data matrices revealed that they are comprised of variable sets of genes (see Fig. S8 at figshare, https://doi.org/10.6084/m9.figshare.6465011). For example, the largest intersection among NT data matrices comprised 207 genes that were shared between all NT matrices except the completeness-based one; similarly, the largest intersection among AA data matrices was 228 genes and was shared between all AA matrices except the completeness-based one (see Fig. S8a and b at figshare, https://doi.org/10.6084/m9.figshare.6465011). Examination of the number of genes overlapping between the NT and AA data matrices for each criterion (see Fig. S8c at figshare, https://doi.org/10.6084/m9.figshare.6465011) showed that three criteria yielded identical or nearly identical NT and AA gene sets. These were completeness (834/834; 100% shared genes; *r_s_* = 1.00, *P < *0.01) (see Fig. S7c at figshare, https://doi.org/10.6084/m9.figshare.6465011), alignment length (829/834; 99.4% shared genes; *r_s_* = 1.00, *P < *0.01) (see Fig. S7f at figshare, https://doi.org/10.6084/m9.figshare.6465011), and the number of variable sites (798/834; 95.7% shared genes; *r_s_* = 0.99, *P < *0.01) (see Fig. S7i at figshare, https://doi.org/10.6084/m9.figshare.6465011). The other two criteria showed greater differences between NT and AA data matrices (average bootstrap value, 667/834; 80.0% shared genes; *r_s_* = 0.78, *P < *0.01 [see Fig. S7l at figshare, https://doi.org/10.6084/m9.figshare.6465011]; treeness/RCV, 644/834; 77.2% shared genes; *r_s_* = 0.72, *P < *0.01 [see Fig. S7o at figshare, https://doi.org/10.6084/m9.figshare.6465011]). However, we note that the NT data matrices always outperformed AA data matrices (see Fig. S6 at figshare, https://doi.org/10.6084/m9.figshare.6465011), suggesting that evaluation of the phylogenetic signal of sequence type is an important parameter in phylogenomic studies.

### A genome-scale phylogeny for the family Aspergillaceae.

**FIG 1:**
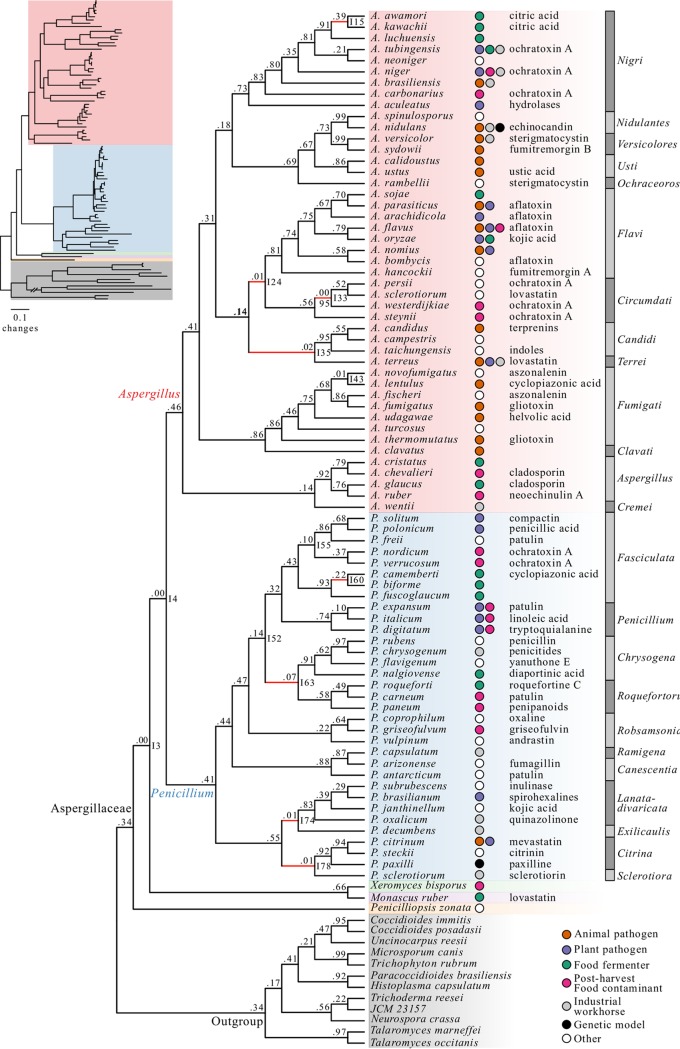
A robust genome-scale phylogeny for the fungal family Aspergillaceae. Different genera are depicted using different-colored boxes: *Aspergillus* is shown in red, *Penicillium* in blue, *Xeromyces* in green, *Monascus* in purple, and *Penicilliopsis* in orange. Different sections within *Aspergillus* and *Penicillium* are depicted with alternating dark gray and gray bars. Internode certainty values are shown below each internode, and bootstrap values are shown above each internode (only bootstrap values lower than 100% are shown). Internode certainty values were calculated using the 1,668 maximum likelihood single-gene trees. Five thousand ultrafast bootstrap replicates were used to determine internode support. Internodes were considered unresolved if they were not present in one or more of the other 35 phylogenies represented in Fig. 2—the branches of these unresolved internodes are drawn in red. Additional incongruent internodes were identified using calculations of IC. The inset depicts the phylogeny with branch lengths corresponding to estimated nucleotide substitutions per site. Colored circles next to species names indicate the lifestyle or utility of the species (i.e., animal pathogen, dark orange; plant pathogen, purple; food fermenter, green; postharvest food contaminant, pink; industrial workhorse, gray; genetic model, black; other, white). Exemplary secondary metabolites produced by different Aspergillaceae species are written to the right of the colored circles.

**FIG 2:**
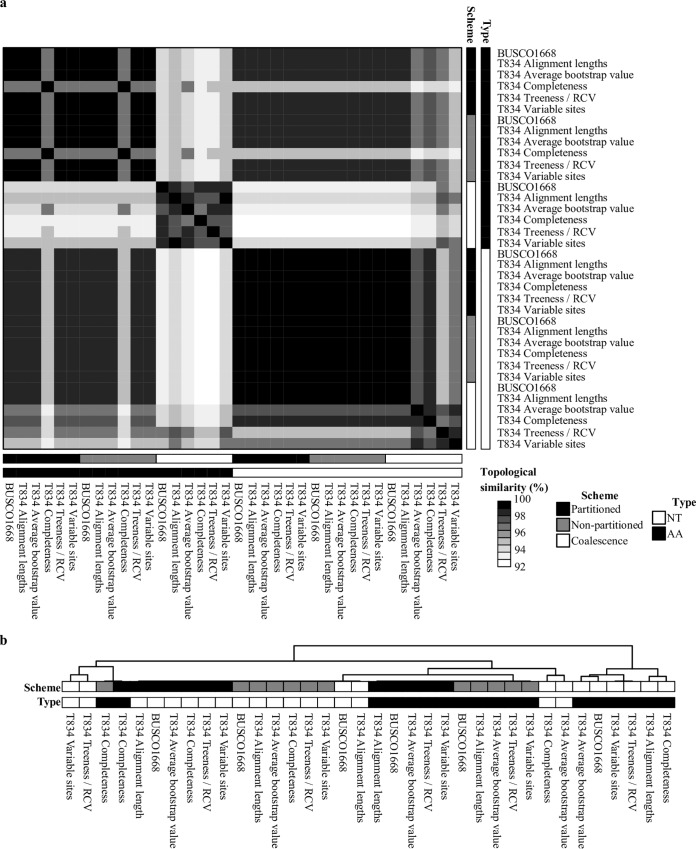
Topological similarity between the 36 phylogenies constructed using 6 different data matrices, 2 different sequence types, and 3 analytical schemes. (a) A heat map depiction of topological similarity between the 36 phylogenies constructed in this study. The 36 phylogenies were inferred from analyses of 2 different sequence types (i.e., protein, depicted in black; nucleotide, depicted in white), 3 different analytical schemes (i.e., partitioned, depicted in black; nonpartitioned, depicted in gray; coalescence, depicted in white), and 6 different matrices (full data matrix, “BUSCO1668,” and 5 subsampled ones, all starting with “T834”; depending on the subsampling strategy, they are identified as “T834 Alignment lengths,” “T834 Average bootstrap value,” “T834 Completeness,” “T834 Treeness/RCV,” and “T834 Variable sites”). (b) Hierarchical clustering based on topological similarity values among the 36 phylogenies.

**FIG 3:**
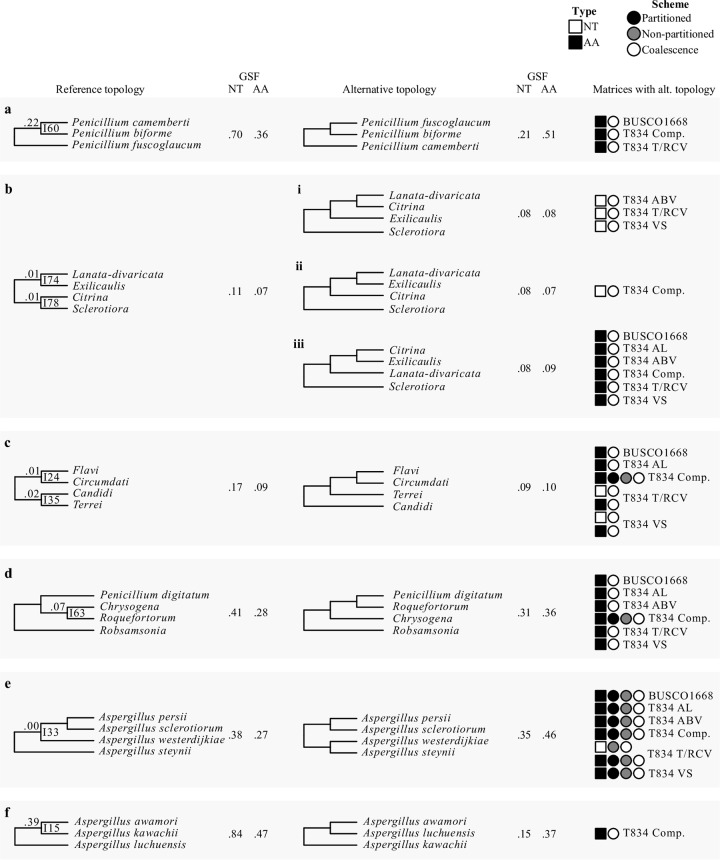
The eight internodes not recovered in all 36 phylogenies. Internode numbers refer to internodes that have at least one conflicting topology among the 36 phylogenetic trees inferred from the full and five subsampled data matrices across three different schemes and two data types. The internode recovered from the analysis of the 1,668-gene nucleotide matrix (Fig. 1) is shown on the left and the conflicting internode(s) on the right. Next to each of the internodes, the nucleotide (NT) and amino acid (AA) gene support frequency (GSF) values are shown. On the far right, the sequence type, scheme, and data matrix characteristics of the phylogenies that support the conflicting internodes are shown. NT and AA sequence types are represented using white and black squares, respectively; partitioned concatenation, nonpartitioned concatenation, and coalescence analytical schemes are depicted as black, gray, or white circles, respectively; and the matrix subset is written next to the symbols.

**FIG 4:**
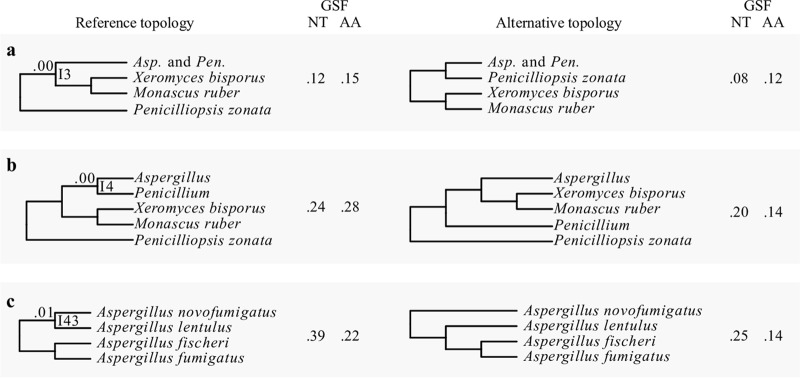
The three internodes recovered in all 36 phylogenies but that exhibit very low internode certainty values. Three bipartitions were recovered by all 36 phylogenies but had internode certainty values below 0.10 (a to c). The internode recovered from the analysis of all 36 phylogenies, including of the 1,668-gene nucleotide matrix (Fig. 1), is shown on the left and the most prevalent, conflicting internode on the right. Next to each of the internodes, the nucleotide (NT) and amino acid (AA) gene support frequency (GSF) values are shown.

NT and AA phylogenomic analyses of the full data matrix and the five subsampled data matrices under three analytical schemes recovered a broadly consistent set of relationships (Fig. 1 to 4). Across all 36 species-level phylogenies, we observed high levels of topological similarity (average topological similarity, 97.2% ± 2.5%; minimum, 92.2%; maximum, 100%) (Fig. 2), with both major genera (*Aspergillus* and *Penicillium*) as well as all sections in *Aspergillus* and *Penicillium* (61, 66) recovered as monophyletic (Fig. 1, 3, and 4). Additionally, all but one internode exhibited absolute UFBoot scores (67); the sole exception was internode 33 (I33), which received 95 UFBoot support (Fig. 1: see also Fig. S9 at figshare, https://doi.org/10.6084/m9.figshare.6465011).

Surprisingly, one taxon previously reported to be part of Aspergillaceae, Basipetospora chlamydospora, was consistently placed among outgroup species (Fig. 1) and may represent a misidentified isolate. To identify the isolate’s true identity, we blasted the nucleotide sequence of *tef1* from the isolate against the “nucleotide collection (nr/nt)” database using MEGABlast (68) on NCBI’s webserver. We found the top three hits were to Podospora anserina (class Sordariomycetes, PODANS_1_19720; E value, 0.0; maximum score, 1,753; percent identity, 91%), Scedosporium apiospermum (class Sordariomycetes, SAPIO_CDS5137; E value, 0.0; maximum score, 1,742; percent identity,: 92%), and Isaria fumosorosea (class Sordariomycetes, ISF_05984; E value, 0.0; maximum score, 1,724; percent identity, 90%). These results make it difficult to ascribe the genome of the misidentified isolate to a specific genus and species but confirm its placement outside Aspergillaceae; we refer to the isolate by its strain identifier, JCM 23157. Thus, our phylogenomic approach can be a powerful tool in establishing the accuracy of the taxonomic information associated with genomic sequence data and, in certain cases, a valuable additional tool for strain identification.

### Examination of the Aspergillaceae phylogeny reveals 11 incongruent bipartitions.

Examination of all 36 species-level phylogenies revealed the existence of 8 (8/78; 10.3%) incongruent bipartitions. Complementary examination of internode certainty (IC), a bipartition-based measure of incongruence, revealed an additional 3/78 (3.8%) bipartitions that displayed very high levels of incongruence at the gene level, raising the total number of incongruent bipartitions to 11 (11/78; 14.1%).

Examination of the eight conflicting bipartitions stemming from the comparison of the 36 phylogenies showed that they were very often associated with data type (NT or AA) and scheme employed (concatenation or coalescence). For example, the first instance of incongruence concerns the identity of the sister species to Penicillium biforme (I60 [Fig. 1 and 3a]); this species is *P. camemberti* in the reference phylogeny, but analyses of the full and two subsampled AA data matrices with coalescence recover instead Penicillium fuscoglaucum. The data type and analytical scheme employed also appear to underlie the second and third instances of incongruence, which concern the placement of sections *Exilicaulis* and *Sclerotiora* (I74 and I78 [Fig. 1 and 3b]); the fourth and fifth instances, which concern relationships among *Aspergillus* sections (I24 and I35 [Fig. 1 and 3c]); and the sixth instance, which concerns relationships among *Penicillium digitatum* and the sections *Chrysogena* and *Roquefortorum* (I63 [Fig. 1 and 3d]). The seventh instance is also associated with data type, but not with the scheme employed; while the reference as well as most subsampled NT matrices supports the Aspergillus persii and Aspergillus sclerotiorum clade as sister to Aspergillus westerdijkiae (I33 [Fig. 1 and 3e]), most AA data matrices recover a conflicting bipartition where Aspergillus steynii is the sister group of A. westerdijkiae. The final instance of incongruence was the least well supported, as 35/36 (97.2%) phylogenies supported Aspergillus kawachii as the sister group to Aspergillus awamori (I15 [Fig. 1 and 3f]), but analysis of one AA subsampled data matrix with coalescence instead recovered Aspergillus luchuensis as the sister group.

For each of these bipartitions (Fig. 3), we examined clustering patterns using multiple correspondence analysis of matrix features (i.e., sequence type and subsampling method) and an analysis scheme among trees that support the reference and alternative topologies (see Fig. S10 at figshare, https://doi.org/10.6084/m9.figshare.6465011). Distinct clustering patterns were observed for I74, I78, and I33 (Fig. 3; also see Fig. S10 at figshare, https://doi.org/10.6084/m9.figshare.6465011). For I74 and I78, there are three alternative, conflicting topologies, with the first two clustering separately from the third (Fig. 3b; also see Fig. S10b at figshare, https://doi.org/10.6084/m9.figshare.6465011). For I33, phylogenies that support the reference and alternative topologies formed distinct clusters (Fig. 3e). Examination of the contribution of variables along the second dimension, which is the one that differentiated variables that supported each topology, revealed that the distinct clustering patterns were driven by sequence type (see Fig. S10g and h at figshare, https://doi.org/10.6084/m9.figshare.6465011). Previous analyses indicated that NT data matrices outperformed AA data matrices regardless of method of inference or the subsampled data matrix used (see Fig. S6 at figshare, https://doi.org/10.6084/m9.figshare.6465011). This suggests that these cases of incongruence may be due to the less robust phylogenetic signal of AAs for the present data set.

Examination of IC values revealed three additional bipartitions with strong signatures for incongruence at the gene level, defined as an IC score lower than 0.10. The first instance concerns the sister taxon to the *Aspergillus* and *Penicillium* clade. Although all 36 phylogenies recover a clade comprised of Xeromyces bisporus and Monascus ruber as the sister group, the IC score for this bipartition is 0.00 (I3 [Fig. 4a]); the most prevalent, conflicting bipartition supports Penicilliopsis zonata as sister to *Aspergillus* and *Penicillium* (Fig. 4a). Similarly, although all 36 phylogenies recover *Penicillium* as sister to *Aspergillus*, the IC score for this bipartition is also 0.00 (I4 [Fig. 4b]); the most prevalent, conflicting bipartition supports *X. bisporus* and *M. ruber* as the sister clade to *Aspergillus* (Fig. 4b). In the third instance, all 36 phylogenies support Aspergillus novofumigatus and Aspergillus lentulus as sister species, but the IC score of this bipartition is 0.01 (I43 [Fig. 4c]); the most prevalent, conflicting bipartition recovers A. lentulus as the sister species to a clade comprised of Aspergillus fumigatus and Aspergillus fischeri (Fig. 4c).

To examine the underlying individual gene support to the resolution of these 11 bipartitions, we examined the phylogenetic signal contributed by each individual gene in the full NT data matrix. In all 11 bipartitions, we found that inferences were robust to single gene outliers with strong phylogenetic signal (see Fig. S11 and File S3 at figshare, https://doi.org/10.6084/m9.figshare.6465011).

To determine if robustly identified internodes were sensitive to potential hidden paralogs, we reevaluated IC in a set of 1,331 genes that passed our hidden paralogy filter. We observed that measurements of IC were very similar between the 1,668- and 1,331-gene NT data sets (*r_s_* = 0.98, *P < *0.01) (see Fig. S12 at figshare, https://doi.org/10.6084/m9.figshare.6465011). Notably, we did not identify any additional internodes with evidence of incongruence. In contrast, examination of IC in the 1,331-gene tree set showed reduced levels of incongruence at I63 (Fig. 3d; IC value using the 1,668-gene data matrix = 0.07, IC value using the 1,331-gene data matrix = 0.10). Although the reduction in incongruence levels is not significant, these results suggest that removal of potential hidden paralogs may provide more accurate measures of IC.

To determine if removal of potential hidden paralogs and the use of different alignment trimming methods influenced inference of the species phylogeny, we reinferred species trees using the three different maximum likelihood approaches across the five data sets, resulting in 25 additional phylogenies ([2 sequence types × 2 BMGE trimming approaches × 3 maximum likelihood schemes × 2 gene data sets of size 1,668 and 1,331] + 1,331-gene data set trimmed using trimAl). Neither the removal of potential hidden paralogs nor the use of different trimming methods altered the topology of the species phylogeny in 21 of the 25 (84%) cases. In the remaining four cases, the topologies recovered conflicted with the species phylogeny in Fig. 1 with respect to an already-identified conflict (Fig. 3 and 4). Specifically, the species phylogeny inferred using coalescence with the 1,668-gene NT matrix trimmed using BMGE_0.7_ inferred the topology discussed in Fig. 3b, subpanel iii, and the 1,668-AA gene matrix trimmed using BMGE_0.5_ and BMGE_0.7_ and the 1,331-NT gene matrix trimmed using BMGE_0.7_ (all analyzed using coalescence) inferred the topology discussed in Fig. 3f.

Finally, to evaluate whether phylogenetic inference using site-homogenous models had any impact on the observed incongruence, we inferred three additional species-level phylogenies using the C40 and C60 mixture models as well as the posterior mean site frequency (PMSF) model. We found that all three models inferred the same topology as the full original amino acid data matrix with a gene-partitioning scheme and site-homogeneous models. These results suggest that our inferences are robust under both site-homogeneous and site-heterogeneous models of phylogenetic inference.

### Incongruence in the Aspergillaceae phylogeny.

Examination of the 11 incongruent bipartitions with respect to their placement on the phylogeny (shallow, i.e., near the tips of the phylogeny, or deeper, i.e., away from the tips and toward the base of the phylogeny) and the amount of conflict (quantified using IC and gene support frequencies [GSF] [Fig. 1; see also File S4 at figshare, https://doi.org/10.6084/m9.figshare.6465011) allowed us to group them into three categories: (i) shallow bipartitions (I15 and I60) with low levels of incongruence, (ii) shallow bipartitions (I33 and I43) with high levels of incongruence, and (iii) deeper bipartitions (I3, I4, I24, I35, I63, I74, and I78) with various levels of incongruence and typically associated with single-taxon long branches.

### (i) Shallow bipartitions with low levels of incongruence.

The two bipartitions that fell into this category, I60 (Fig. 3a) and I15 (Fig. 3f), exhibited low levels of incongruence among closely related taxa. For I60, the reference bipartition was observed in 33/36 phylogenies and had an IC score of 0.22 and GSF_NT_ and GSF_AA_ scores of 0.70 and 0.21, respectively. Similarly, the reference bipartition for I15 was observed in 35/36 phylogenies and had an IC score of 0.39 and GSF_NT_ and GSF_AA_ scores of 0.84 and 0.47, respectively. Notably, the GSF_NT_ scores were substantially higher for the reference bipartitions in both of these cases.

### (ii) Shallow bipartitions with high levels of incongruence.

The two shallow bipartitions, I33 (Fig. 3e) and I43 (Fig. 4c), in this category exhibited high levels of incongruence among closely related taxa. For I33, the reference bipartition was observed in 16/36 phylogenies (44.4%) and had an IC score of 0.00 and GSF_NT_ and GSF_AA_ scores of 0.38 and 0.27, respectively. The reference bipartition for I43 was observed in all 36 phylogenies and had an IC score of 0.01 and GSF_NT_ and GSF_AA_ scores of 0.39 and 0.22, respectively. Notably, in both cases, substantial fractions of genes supported both the reference and the conflicting bipartitions, with both the GSF_NT_ and GSF_AA_ scores of each pair of bipartitions being almost always higher than 0.2.

### (iii) Deeper bipartitions often associated with single-taxon long branches.

The seven bipartitions in this category were I74 and I78 (Fig. 3b), I24 and I35 (Fig. 3c), I63 (Fig. 3d), I3 (Fig. 4a), and I4 (Fig. 4b). All of them are located deeper in the tree, and most involve single taxa with long terminal branches (Fig. 1). The reference bipartitions for internodes I74 and I78, which concern relationships among the sections *Lanata-divaricata*, *Exilicaulis*, *Citrina*, and *Sclerotiora* were observed in 26/36 (72.2%) phylogenies; the remaining 10/36 (27.8%) phylogenies recovered three alternative, conflicting bipartitions. Both reference bipartitions had IC scores of 0.01 and GSF_NT_ and GSF_AA_ scores of 0.11 and 0.07, respectively. The reference bipartitions for internodes I24 and I35, which concern the placement of Aspergillus terreus, the single taxon representative of section *Terrei*, were observed in 27/36 (75.0%) phylogenies and had IC scores of 0.01 and 0.02 and GSF_NT_ and GSF_AA_ scores of 0.17 and 0.09, respectively. The reference bipartition I63, which involved the placement of *Penicillium digitatum*, a member of section *Penicillium*, was observed in 28/36 (77.8%) phylogenies and had an IC score of 0.07 and GSF_NT_ and GSF_AA_ scores of 0.41 and 0.28, respectively. Notably, the alternative topology recovers section *Penicillium* as polyphyletic. We also noted that the IC score for this bipartition in the hidden paralogy-filtered 1,331-gene data set increased to 0.10, suggesting that hidden paralogy may be a contributing factor to the observed incongruence at this internode. Finally, the reference bipartitions I3 and I4 (Fig. 4), which concern the identity of the sister taxon of *Aspergillus* and *Penicillium* (I3) and the identity of the sister taxon of *Aspergillus* (I4), were found in all 36 phylogenies but both had IC values of 0.00. For I3, GSF_NT_ and GSF_AA_ scores were 0.12 and 0.15, respectively. For I4, GSF_NT_ and GSF_AA_ scores were 0.24 and 0.28, respectively. Last, the reference bipartition I52 was observed in all 36 phylogenies and had an IC score of 0.14 using the 1,668-gene data set trimmed using trimAl but an IC score of 0.07 in the 1,668-gene data set trimmed by BMGE_0.5_.

### Topology tests.

**FIG 5:**
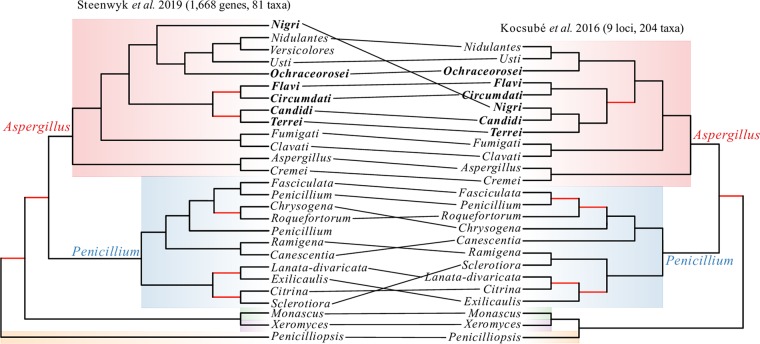
A visual comparison of the differences between the phylogeny reported in this study and the phylogeny reported in the work of Kocsubé et al. (61). Tanglegram between the section-level phylogeny presented in this study (left) and the section-level phylogeny presented by Kocsubé et al. (61) (right). The key differences between the two phylogenies lie in the placements of sections *Nigri*, *Ramigena*, and *Canescentia*. Species in bold belong to narrow *Aspergillus*, and red branches represent bipartitions that are not robustly supported in each study.

**FIG 6:**
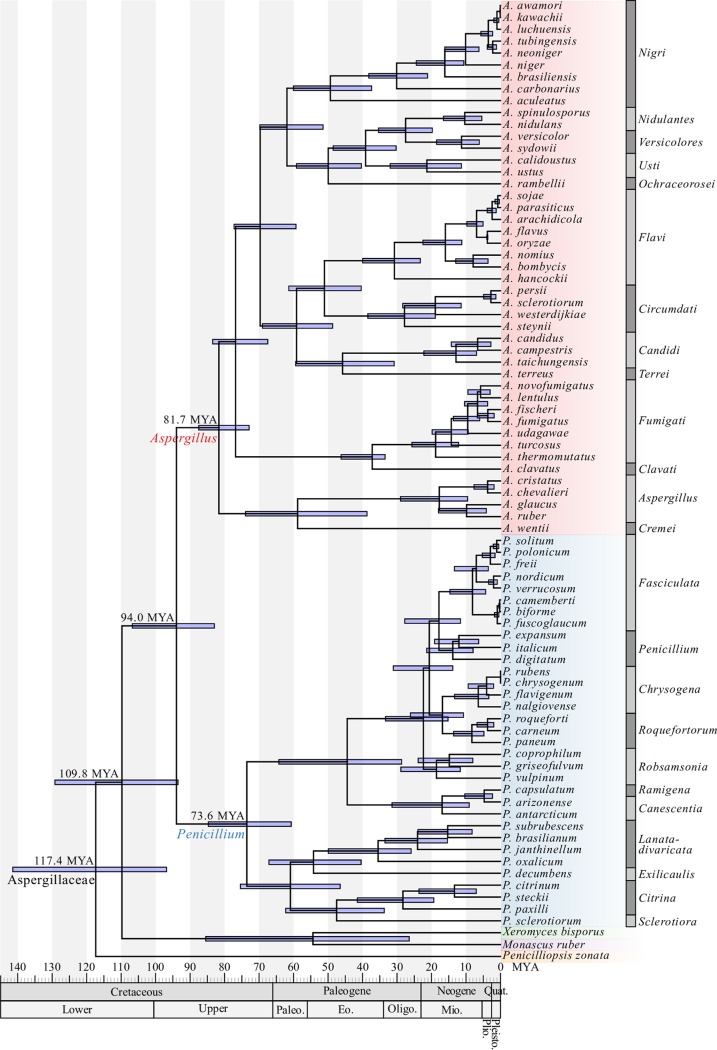
A molecular time tree for the family Aspergillaceae. Blue boxes around each internode correspond to 95% divergence time confidence intervals for each branch of the Aspergillaceae phylogeny. For reference, the geologic time scale is shown right below the phylogeny. Different genera are depicted using different-colored boxes; *Aspergillus* is shown in red, *Penicillium* in blue, *Xeromyces* in green, *Monascus* in purple, and *Penicilliopsis* in orange. Different sections within *Aspergillus* and *Penicillium* are depicted with alternating dark gray and gray bars. Dating estimates were calibrated using the following constraints: origin of Aspergillaceae (I2; 50 to 146 million years ago [mya]), origin of *Aspergillus* (I5; 43 to 85 mya), the A. flavus and *A. oryzae* split (I30; 3.68 to 3.99 mya), and the A. fumigatus and *A. clavatus* split (I38; 35 to 39 mya); all constraints were obtained from TimeTree (91).

The phylogeny of the genera *Aspergillus* and *Penicillium* has been a topic of debate. Our topology supports the reciprocal monophyly of *Aspergillus* and *Penicillium* and rejects the monophyly of narrow *Aspergillus*. Both of these results are consistent with some previous studies (61) (see Fig. 6) but in contrast to other previous studies, which recovered a topology where *Penicillium* is sister to section *Nidulantes* within *Aspergillus* and narrow *Aspergillus* (sections *Nigri*, *Ochraceorosei*, *Flavi*, *Circumdati*, *Candidi*, and *Terrei*) is monophyletic (43, 62). To further evaluate both of these hypotheses, we conducted separate topology constraint analyses using the Shimodaira-Hasegawa test (69) and the approximately unbiased tests (70). Both tests rejected the constrained topologies (Table 1; *P* value < 0.001 for all tests), providing further support that *Aspergillus* and *Penicillium* are reciprocally monophyletic and that narrow *Aspergillus* is not monophyletic (Fig. 5). Our results reveal the potential of phylogenomic approaches to resolve longstanding debates of incongruence, especially ones associated with deep internodes.

**TABLE 1:** Topology tests reject the sister group relationship of genus *Penicillium* and *Aspergillus* section *Nidulantes* as well as the monophyly of narrow *Aspergillus*

Constrained topology	Likelihood of tree		Difference in log likelihood	*P* value by test:	
	Unconstrained	Constrained		Shimodaira-Hasegawa	Approximately unbiased
Sister group relationship of genus *Penicillium* and *Aspergillus* section *Nidulantes*	−99,617,175.719	−99,767,653.909	150,478.190	<0.001	<0.001
Monophyly of narrow *Aspergillus*	−99,617,175.719	−99,730,789.937	113,614.218	<0.001	<0.001

### A geological timeline for the evolutionary diversification of the Aspergillaceae family.

To estimate the evolutionary diversification among Aspergillaceae, we subsampled the 1,668-gene matrix for high-quality genes with “clock-like” rates of evolution by examining degree of violation of a molecular clock (DVMC) (71) values among single-gene trees. Examination of the DVMC values facilitated the identification of a tractable set of high-quality genes for relaxed molecular clock analyses (see Fig. S13 and File S5 at figshare, https://doi.org/10.6084/m9.figshare.6465011). We found that Aspergillaceae originated 117.4 (95% CI, 141.5 to 96.9) mya during the Cretaceous period (Fig. 6; see also File S6 at figshare, https://doi.org/10.6084/m9.figshare.6465011). We found that the common ancestor of *Aspergillus* and *Penicillium* split from the *X. bisporus* and *M. ruber* clade shortly thereafter, approximately 109.8 (95% CI, 129.3 to 93.5) mya. We also found that the genera *Aspergillus* and *Penicillium* split 94.0 (95% CI, 106.8 to 83.0) mya, with the last common ancestor of *Aspergillus* originating approximately 81.7 mya (95% CI, 87.5 to 72.9) and the last common ancestor of *Penicillium* originating approximately 73.6 mya (95% CI, 84.8 to 60.7).

Among *Aspergillus* sections, section *Nigri*, which includes the industrial workhorse Aspergillus niger, originated 49.4 (95% CI, 60.1 to 37.4) mya. Section *Flavi*, which includes the food fermenters *A. oryzae* and Aspergillus sojae and the toxin-producing, postharvest food contaminant and opportunistic animal and plant pathogen A. flavus, originated 30.8 (95% CI, 40.0 to 23.3) mya. Additionally, section *Fumigati*, which includes the opportunistic human pathogen A. fumigatus, originated 18.8 (95% CI, 25.7 to 12.2) mya. Among *Penicillium* sections, section *Fasciculata*, which contains Camembert and Brie cheese producer *P. camemberti* and the ochratoxin A producer, *P. verrucosum*, originated 8.1 (95% CI, 14.7 to 4.3) mya. Section *Chrysogena*, which includes the antibiotic penicillin-producing species P. chrysogenum, originated 6.5 (95% CI, 13.3 to 3.4) mya. Additionally, section *Citrina*, which contains *P. citrinum*, from which the first statin was isolated and which is commonly associated with moldy citrus fruits (72), originated 28.3 (95% CI, 41.5 to 19.3) mya.

Finally, our analysis also provides estimates of the origins of various iconic pairs of species within *Aspergillus* and *Penicillium*. For example, among *Aspergillus* species pairs, we estimate that A. fumigatus and the closest relative with a sequenced genome, *A. fischeri* (73), diverged 3.7 (95% CI, 6.7 to 1.9) mya and Aspergillus flavus and the domesticated counterpart, *A. oryzae* (8), diverged 3.8 (95% CI, 4.0 to 3.7) mya. Among *Penicillium* species pairs, we estimate *P. camemberti*, which contributes to cheese production, to have diverged from its sister species and cheese contaminant *P. biforme* (74) approximately 0.3 (95% CI, 0.5 to 0.1) mya. Finally, we estimate that *P. roqueforti*, another species that contributes to cheese production, diverged from its close relative *P. carneum* (74) 3.8 (95% CI, 6.8 to 2.0) mya.

## DISCUSSION

Our analyses provide a robust evaluation of the evolutionary relationships and diversification among Aspergillaceae, a family of biotechnologically and medically significant fungi. We scrutinized our proposed reference phylogeny (Fig. 1) against 35 other phylogenies recovered using all possible combinations of six multigene data matrices (full or subsamples thereof), three maximum likelihood schemes, and two sequence types and complemented this analysis with bipartitioning-based measures of support (Fig. 1 and 2). We also examined the robustness of our proposed reference phylogeny to different sequence alignment trimming methods, the removal of potential hidden paralogs, and site-heterogeneous substitution models. Through these analyses, we found that 11/78 (14.1%) bipartitions were incongruent (Fig. 3 and 4) and explored the characteristics as well as sources of these instances of incongruence. Finally, we placed the evolution and diversification of Aspergillaceae in the context of geological time.

Comparison of our 81-taxon, 1,668-gene phylogeny to a previous one based on a maximum likelihood analysis of 9 loci for 204 Aspergillaceae species (61) suggests that our analyses identified and strongly supported several new relationships and resolved previously poorly supported bipartitions (Fig. 1 and 5). The robust resolution of our phylogeny is likely due to the very large size of our data matrix, both in terms of genes and in terms of taxa. For example, the placement of *Aspergillus* section *Nigri* has been unstable in previous phylogenomic analyses (see Fig. S1 at figshare, https://doi.org/10.6084/m9.figshare.6465011) (23, 48, 49), but our denser sampling of taxa in this section as well as inclusion of representative taxa from sections *Nidulantes*, *Versicolores*, *Usti*, and *Ochraceorosei* now provides strong support for the sister relationship of the *Aspergillus* section *Nigri* to sections *Nidulantes*, *Versicolores*, *Usti*, and *Ochraceorosei* (Fig. 1).

However, our analysis also identified several relationships that exhibit high levels of incongruence (Fig. 3 and 4). In general, gene tree incongruence can stem from biological or analytical factors (46, 59). Biological processes such as incomplete lineage sorting (ILS) (75), hybridization (76), gene duplication and subsequent loss (77), horizontal gene transfer (78), and natural selection (79, 80) can cause the histories of genes to differ from one another and from the species phylogeny. Importantly, although the expected patterns of incongruence will be different for each factor and depend on a number of parameters, the observed patterns of conflict in each of the 11 cases of incongruence in the Aspergillaceae phylogeny can yield insights and allow the formation of hypotheses about the potential drivers in each case. For example, ILS often results in relatively low levels of incongruence; for instance, examination of the human, chimp, and gorilla genomes has showed that 20 to 25% of the gene histories differ from the species phylogeny (81, 82). In contrast, recent hybridization is expected to typically produce much higher levels of incongruence due to rampant sequence similarity among large amounts of genomic content; for instance, examination of *Heliconius* butterfly genomes revealed incongruence levels higher than 40% (83).

Additionally, analytical factors such as model choice (51), taxon sampling (84, 85), hidden paralogy (86, 87), and alignment strategy (88) can lead to erroneous inference of gene histories. Perhaps the best-known instance of incongruence stemming from analytical factors is what is known as “long branch attraction,” namely, the situation where highly divergent taxa, i.e., the ones with the longest branches in the phylogeny, will often artifactually group with other long branches (89). Examination of the effects of removal of potential hidden paralogs and different alignment trimming strategies showed that these analytical factors did not substantially contribute to the observed incongruence (see Fig. S12 at figshare, https://doi.org/10.6084/m9.figshare.6465011).

Examination of the patterns of incongruence in the Aspergillaceae phylogeny allows us not only to group the 11 incongruent internodes with respect to their patterns of conflict but also to postulate putative drivers of the observed incongruence. For example, both I15 and I60 are shallow internodes exhibiting low levels of incongruence, suggesting that one likely driver of the observed incongruence is ILS. For bipartition I60, we note that our analysis does not include Penicillium commune, the undomesticated relative of *P. camemberti* (90), which is likely to be key in further understanding the observed incongruence. In contrast, the shallow internodes I33 and I43 exhibit much higher levels of incongruence that are most likely to be the result of processes such as hybridization or repeated introgression. Finally, the remaining seven incongruent internodes (I3, I4, I24, I35, I63, I74, and I78) exhibit various levels of incongruence and are typically associated with single-taxon long branches (Fig. 1, 3, and 4), implicating taxon sampling as a likely driver of the observed incongruence. Given that inclusion of additional taxa robustly resolved the previously ambiguous placement of the long-branched *Aspergillus* section *Nigri* (see discussion above) as well as of other contentious branches of the fungal tree of life, such as the placement of the budding yeast family *Ascoideaceae* (59, 60), we predict that additional sampling of taxa that break up the long branches associated with these seven internodes will lead to their robust resolution. Last, the IC value of internode I63 following removal of hidden paralogs marginally increased, suggesting that incongruence at this internode may also be associated with hidden paralogs.

Notably, the topology of our phylogeny was able to resolve two contentious issues that emerged from analyses of data matrices containing a few genes (61, 62) and that are important for taxonomic relationships within the family. Specifically, our phylogenetic analyses rejected the sister group relationship of genus *Penicillium* and *Aspergillus* section *Nidulantes* as well as the monophyly of a group of *Aspergillus* sections that are referred to as narrow *Aspergillus* (Table 1; *P* value < 0.001 for all tests). Instead, our phylogeny shows that the genera *Aspergillus* and *Penicillium* are reciprocally monophyletic. These results are consistent with the current nomenclature proposed by the International Commission of *Penicillium* and *Aspergillus* (https://www.aspergilluspenicillium.org/) and inconsistent with the phylogenetic arguments put forward in proposals for taxonomic revision (62). However, it should be noted that our study did not include representatives of the genera *Phialosimplex* and *Polypaecilum*, which lack known asexual stages and appear to be placed within the genus *Aspergillus* (61, 62). *Basipetospora* species also lack known asexual stages and are also placed within *Aspergillus* (61, 62); unfortunately, the sole genome sequenced from this genus, JCM 23157, appears to be a contaminant from the class Sordariomycetes (Fig. 1).

Finally, our relaxed molecular clock analysis of the Aspergillaceae phylogeny provides a robust and comprehensive time scale for the evolution of Aspergillaceae and its two large genera, *Aspergillus* and *Penicillium* (Fig. 6), filling a gap in the literature. Previous molecular clock studies provided estimates for only four internodes, mostly within the genus *Aspergillus* (91–99), and yielded much broader time intervals. For example, the previous estimate for the origin of Aspergillaceae spanned nearly 100 mya (50 to 146 mya [92–94]) while our data set and analysis provided a much narrower range of 44.7 mya (mean, 117.4; 95% CI, 141.5 to 96.9). Notably, the estimated origins of genera *Aspergillus* (∼81.7 mya) and *Penicillium* (∼73.6 mya) appear to be comparable to those of other well-known filamentous fungal genera, such as *Fusarium*, whose date of origin has been estimated at ∼91.3 mya (100, 101).

### Conclusion.

Fungi from Aspergillaceae have diverse ecologies and play significant roles in biotechnology and medicine. Although most of the 81 genomes from Aspergillaceae are skewed toward two iconic genera, *Aspergillus* and *Penicillium*, and do not fully reflect the diversity of the family, they do provide a unique opportunity to examine the evolutionary history of these important fungi using a phylogenomic approach. Our scrutiny of the Aspergillaceae phylogeny, from the Cretaceous to the present, provides strong support for most relationships within the family as well as identifying a few that deserve further examination. Our results suggest that the observed incongruence is likely associated with diverse processes such as incomplete lineage sorting, hybridization, and introgression, as well as with analytical issues associated with poor taxon sampling. Our elucidation of the tempo and pattern of the evolutionary history of Aspergillaceae aids efforts to develop a robust taxonomic nomenclature for the family and provides a robust phylogenetic and temporal framework for investigation of the evolution of pathogenesis, secondary metabolism, and ecology of this diverse and important fungal family.

## MATERIALS AND METHODS

### Genome sequencing and assembly.

Mycelia were grown on potato dextrose agar for 72 h before lyophilization. Lyophilized mycelia were lysed by grinding in liquid nitrogen and suspension in extraction buffer (100 mM Tris-HCl, pH 8, 250 mM NaCl, 50 mM EDTA, and 1% SDS). Genomic DNA was isolated from the lysate with a phenol-chloroform extraction followed by an ethanol precipitation.

DNA was sequenced with both paired-end and mate-pair strategies to generate a high-quality genome assembly. Paired-end libraries and mate-pair libraries were constructed at the Genomics Services Lab at HudsonAlpha (Huntsville, AL) and sequenced on an Illumina HiSeq X sequencer. Paired-end libraries were constructed with the Illumina TruSeq DNA kit, and mate-pair libraries were constructed with the Illumina Nextera mate-pair library kit targeting an insert size of 4 kb. In total, 63 million paired-end reads and 105 million mate-pair reads, each of which was 150 bp in length, were generated.

The Aspergillus spinulosporus genome was assembled using the iWGS pipeline (102). Paired-end and mate-pair reads were assembled with SPAdes, version 3.6.2 (103), using optimal k-mer lengths chosen using KmerGenie, version 1.6982 (104), and evaluated with QUAST, version 3.2 (105). The resulting assembly is 33.8 Mb in size with an *N*_50_ of 939 kb.

### Data collection and quality assessment.

To collect a comprehensive set of genomes representative of Aspergillaceae, we used “Aspergillaceae” as a search term in NCBI’s Taxonomy Browser and downloaded a representative genome from every species that had a sequenced genome as of 5 February 5 2018. We next confirmed that each species belonged to Aspergillaceae according to previous literature reports (23, 66). Since the goal of our study was to examine the evolutionary history of fungi in the family Aspergillaceae, we did not include genomes from well-known genera that belong to other families in the order Eurotiales (e.g., the genus *Talaromyces* from the family *Trichocomaceae*) (1), except as outgroups. Altogether, 80 publicly available genomes and 1 newly sequenced genome spanning 5 genera (45 *Aspergillus* species; 33 *Penicillium* species; one *Xeromyces* species; one *Monascus* species; and one *Penicilliopsis* species) from the family Aspergillaceae were collected (see File S1 at figshare, https://doi.org/10.6084/m9.figshare.6465011). We also retrieved an additional 12 fungal genomes from representative species in the order Eurotiales but outside the family Aspergillaceae to use as outgroups.

To determine if the genomes contained gene sets of sufficient quality for use in phylogenomic analyses, we examined their gene set completeness using Benchmarking Universal Single-Copy Orthologs (BUSCO), version 2.0.1 (106) (see Fig. S2 at figshare, https://doi.org/10.6084/m9.figshare.6465011). In brief, BUSCO uses a consensus sequence built from hidden Markov models derived from 50 different fungal species using HMMER, version 3.1b2 (107), as a query in tBLASTn (108, 109) to search an individual genome for 3,156 predefined orthologs (referred to as BUSCO genes) from the Pezizomycotina database (creation date 13 February 2016) available from OrthoDB, version 9 (65). To determine the copy number and completeness of each BUSCO gene in a genome, gene structure is predicted using AUGUSTUS, version 2.5.5 (110), with default parameters, from the nucleotide coordinates of putative genes identified using BLAST and then aligned to the HMM alignment of the same BUSCO gene. Genes are considered “single copy” if there is only one complete predicted gene present in the genome, “duplicated” if there are two or more complete predicted genes for one BUSCO gene, “fragmented” if the predicted gene is shorter than 95% of the aligned sequence lengths from the 50 different fungal species, and “missing” if there is no predicted gene.

### Phylogenomic data matrix construction.

In addition to their utility as a measure of genome completeness, BUSCO genes have also proven to be useful markers for phylogenomic inference (106) and have been successfully used in phylogenomic studies of clades spanning the tree of life, such as insects (111) and budding yeasts (55, 60). To infer evolutionary relationships, we constructed nucleotide (NT) and amino acid (AA) versions of a data matrix comprised of the aligned and trimmed sequences of numerous BUSCO genes (see Fig. S3 at figshare, https://doi.org/10.6084/m9.figshare.6465011). To construct this data matrix, we first used the BUSCO output summary files to identify orthologous single-copy BUSCO genes with >50% taxon occupancy (i.e., greater than 47/93 taxa have the BUSCO gene present in their genome); 3,138 (99.4%) BUSCO genes met this criterion. For each BUSCO gene, we next created individual AA fasta files by combining sequences across all taxa that have the BUSCO gene present. For each gene individually, we aligned the sequences in the AA fasta file using Mafft, version 7.294b (112), with the BLOSUM62 matrix of substitutions (113), a gap penalty of 1.0, 1,000 maximum iterations, and the “genafpair” parameter. To create a codon-based alignment, we used a custom Python, version 3.5.2 (https://www.python.org/), script using BioPython, version 1.7 (114), to thread codons onto the AA alignment. The NT and AA sequences were then individually trimmed using trimAl, version 1.4 (115), with the “automated1” parameter. To remove potentially spuriously aligned sequences, we removed BUSCO genes whose sequence lengths were less than 50% of the untrimmed length in either the NT or AA sequences, resulting in 1,773 (56.2%) BUSCO genes. Last, we removed BUSCO genes whose trimmed sequence lengths were too short (defined as genes whose alignment length was less than or equal to 167 amino acids and 501 nucleotides), resulting in 1,668 (52.9%) BUSCO genes. The NT and AA alignments of these 1,668 BUSCO genes were then concatenated into the full 1,668-gene NT and AA versions of the phylogenomic data matrix.

To examine the stability of inferred relationships across all taxa, we constructed additional NT and AA data matrices by subsampling genes from the 1,668-gene data matrix that harbor signatures of strong phylogenetic signal. More specifically, we used 5 measures associated with strong phylogenetic signal (116) to create 5 additional data matrices (1 data matrix per measure) comprised of the top-scoring 834 (50%) genes for NTs and AAs (see Fig. S4 at figshare, https://doi.org/10.6084/m9.figshare.6465011). These five measures were: alignment length, average bootstrap value, taxon completeness, treeness/relative composition variability (RCV) (117), and the number of variable sites. We calculated each measure with custom Python scripts using BioPython. Treeness/RCV was calculated using the following formula:

TreenessRCV=∑u=1blult∑i=1c∑j=1n|cij−ci¯|s⋅n

where *l_u_* refers to the internal branch length of the *u*th branch (of *b* internal branches), *l_t_* refers to total tree length, *c* is the number of different characters per sequence type (4 for nucleotides and 20 for amino acids), *n* is the number of taxa in the alignment, *c_ij_* refers to the number of *i*th *c* characters for the *j*th taxon, $\bar{c_{i}}$ refers to the average number of the *i*th *c* character across *n* taxa, and *s* refers to the total number of sites in the alignment. Altogether, we constructed a total of 12 data matrices (one 1,668-gene NT data matrix, one 1,668-gene AA data matrix, five NT subsample data matrices, and five AA subsample data matrices).

### Maximum likelihood phylogenetic analyses.

We implemented a maximum likelihood framework to infer evolutionary relationships among taxa for each of the 1,668 single genes and each of the 12 data matrices separately. For inferences made using either the 1,668- or 834-gene data matrix, we used three different analytical schemes: concatenation with gene-based partitioning, concatenation without partitioning, and gene-based coalescence (46, 118–120). All phylogenetic trees were built using IQ-TREE, version 1.6.1 (121). In each case, we determined the best model for each single gene or partition using the “-m TEST” and “-mset raxml” parameters, which automatically estimate the best-fitting model of substitutions according to their Bayesian information criterion values for either nucleotides or amino acids (122) for those models shared by RAxML (123) and IQ-TREE.

We first examined the inferred best-fitting models across all single-gene trees. Among NT genes, the best-fitting model for 1,643 genes was a general time-reversible model with unequal rates and unequal base frequencies with discrete gamma models, “GTR+G4” (124–126), and for the remaining 25 genes was a general time-reversible model with invariable sites plus discrete gamma models, “GTR+I+G4” (126, 127) (see Fig. S5a at figshare, https://doi.org/10.6084/m9.figshare.6465011). Among AA genes, the best-fitting model for 643 genes was the JTT model with invariable sites plus discrete gamma models, “JTT+I+G4” (127, 128); for 362 genes was the LG model with invariable sites and discrete gamma models, “LG+I+G4” (127, 129); for 225 genes was the JTT model with invariable sites, empirical AA frequencies, and discrete gamma models, “JTT+F+I+G4” (127, 128); and for 153 genes was the JTTDCMut model with invariable sites and discrete gamma models, “JTTDCMut+I+G4” (127, 130) (see Fig. S5b at figshare, https://doi.org/10.6084/m9.figshare.6465011). We used IQ-TREE for downstream analysis because a recent study using diverse empirical phylogenomic data matrices showed that it is a top-performing software program (131).

To reconstruct the phylogeny of Aspergillaceae using a partitioned scheme where each gene has its own model of sequence substitution and rate heterogeneity across site parameters for any given data matrix, we created an additional input file describing these and gene boundary parameters. More specifically, we created a nexus-format partition file that was used as input with the “-spp” parameter, which allows each gene partition in the data matrix to have its set of evolutionary rates (132). To increase the number of candidate trees used during maximum likelihood search, we changed the “-nbest” parameter from the default value of 5 to 10. Last, we conducted 5 independent searches for the maximum likelihood topology using 5 distinct seeds specified with the “-seed” parameter and chose the search with the best log-likelihood score. We used the phylogeny inferred using a partitioned scheme on the full NT data matrix as the reference one for all subsequent comparisons (Fig. 1).

To infer the phylogeny of Aspergillaceae using a nonpartitioned scheme, we used a single model of sequence substitution and rate heterogeneity across sites for the entire matrix. To save computation time, the most appropriate single model was determined by counting which best-fitting model was most commonly observed across single-gene trees. The most commonly observed model was “GTR+F+I+G4” (127, 133), which was favored in 1,643/1,668 (98.5%) of single genes, and “JTT+I+G4” (127, 128), which was favored in 643/1,668 (38.5%) of single genes, for nucleotides and amino acids, respectively (see Fig. S5 at figshare, https://doi.org/10.6084/m9.figshare.6465011). In each analysis, the chosen model was specified using the “-m” parameter.

To reconstruct the phylogeny of Aspergillaceae using coalescence, a method that estimates species phylogeny from single-gene trees under the multispecies coalescent (119), we combined all Newick (134, 135) formatted single-gene trees inferred using their best-fitting models into a single file. The resulting file was used as input to ASTRAL-II, version 4.10.12 (120), with default parameters.

To evaluate support for single-gene trees and for the reference phylogeny (Fig. 1), we used the ultrafast bootstrap approximation approach (UFBoot) (67), an accurate and faster alternative to the classic bootstrap approach. To implement UFBoot for the NT 1,668-gene data matrix and single-gene trees, we used the “-bb” option in IQ-TREE with 5,000 and 2,000 ultrafast bootstrap replicates, respectively.

To reconstruct the phylogeny of Aspergillaceae using site-heterogeneous models or approximations thereof, we inferred species-level phylogenies using the C40 and C60 mixture models (136) as well as the posterior mean site frequency (PMSF) model (137). More specifically, we implemented this approach in a gene-partitioned manner using an edge-linked proportional partition model and increased our search of candidate trees from 5 to 10 using the “-nbest” parameter. For the C40 and C60 models, we used each respective mixture model to infer the phylogeny. For the PMSF model, we estimated the mixture model parameters from which the site-specific frequency profile of the PMSF model is inferred using as our guide tree the maximum likelihood phylogeny inferred using the full amino acid matrix under a gene-partitioned scheme.

### Evaluating topological support.

To identify and quantify incongruence, we used two approaches. In the first approach, we compared the 36 topologies inferred from the full 1,668-gene NT and AA data matrices and five additional 834-gene data matrices (constructed by selecting the genes that have the highest scores in five measures previously shown to be associated with strong phylogenetic signal; see above) using three different maximum likelihood schemes (i.e., gene partitioned, nonpartitioned, and coalescence) and identified all incongruent bipartitions between the reference phylogeny (Fig. 1) and the other 35. In the second approach, we scrutinized each bipartition in the reference phylogeny using measures of internode certainty (IC) measures for complete and partial single-gene trees (45, 63, 64). To better understand single gene support among conflicting bipartitions, we calculated gene-wise log-likelihood scores (GLS) (59) and gene support frequencies (GSF) for the reference and alternative topologies at conflicting bipartitions.

**(i) Identifying internodes with conflict across subsampled data matrices.** To identify incongruent bipartitions between the reference phylogeny and the other 35 phylogenies, we first combined the 36 generated phylogenetic trees into a single file. We next evaluated the support of all bipartitions in the reference topology among the other 35 phylogenies using the “-z” option in RAxML. Any bipartition in the reference phylogeny that was not present in the rest was considered incongruent; each conflicting bipartition was identified through manual examination of the conflicting phylogenies. To determine if sequence type, subsampling method, or maximum likelihood scheme was contributing to differences in observed topologies among conflicting internodes, we conducted multiple correspondence analysis of these features among the 36 phylogenies and visualized results using R, version 3.3.2 (138), packages FactoMineR, version 1.40 (139), and factoextra, version 1.0.5 (140).

**(ii) Identifying internodes with conflict across the 1,668 gene trees.** To examine the presence and degree of support of conflicting bipartitions, we calculated the internode certainty (45, 63, 64, 141) of all internodes in the reference phylogeny (Fig. 1) using the 1,668-gene trees as input. In general, IC scores near 0 indicate that there is near-equal support for an alternative, conflicting bipartition among a set of trees compared to a given bipartition present in the reference topology, which is indicative of high conflict. Therefore, we investigated incongruence in all internodes in the reference phylogeny (Fig. 1) that exhibited IC scores lower than 0.1. To calculate IC values for each bipartition for the reference phylogeny, we created a file with all 1,668 complete and partial single-gene trees. The resulting file of gene trees, specified with the “-z” parameter in RAxML, was used to calculate IC values using the “-f i” argument. The topology was specified with the “-t” parameter. Last, we used the Lossless corrected IC scoring scheme, which corrects for variation in taxon number across single-gene trees (63). We also used these IC values to inform which data type (NT or AA) provided the strongest signal for the given set of taxa and sequences. We observed that NT data consistently exhibited higher IC scores than AA data (hence our decision to use the topology inferred from the full NT data matrix using a gene-partitioned scheme—shown in Fig. 1—as the “reference” topology in all downstream analyses).

**(iii) Examining gene-wise log-likelihood scores for incongruent internodes.** To determine the per-gene distribution of phylogenetic signal supporting a bipartition in the reference phylogeny or a conflicting bipartition, we calculated gene-wise log-likelihood scores (GLS) (59) using the NT data matrix. We chose to calculate GLS using the NT data matrix because distributions of IC values from phylogenies inferred using NT data had consistently higher IC values across schemes and data matrices (see Fig. S6 at figshare, https://doi.org/10.6084/m9.figshare.6465011). To do so, we used functions available in IQ-TREE. More specifically, we inputted a phylogeny with the reference or alternative topology using the “-te” parameter and informed IQ-TREE of gene boundaries, their corresponding models, and optimal rate heterogeneity parameters in the full 1,668-gene data matrix using the “-spp” parameter. Last, we specified that partition log-likelihoods be outputted using the “-wpl” parameter. To determine if a gene provided greater support for the reference or alternative bipartition, we calculated the difference in GLS (ΔGLS) using the following formula:

$${\text{ΔGLS}}_{i}=\ln L{\left(G_{i}\right)}_{\text{ref}}-\ln L{\left(G_{i}\right)}_{\text{alt}}$$

where ln *L*(*G_i_*)_ref_ and ln *L*(*G_i_*)_alt_ represent the log-likelihood values for the reference and alternative topologies for gene *G_i_*, respectively. Thus, values greater than 0 reflect genes in favor of the reference bipartition, values lower than 0 reflect genes in favor of the alternative bipartition, and values of 0 reflect equal support between the reference and alternative bipartitions.

**(iv) Calculating gene support frequencies for reference and conflicting bipartitions.** We next examined support for bipartitions in the reference topology as well as for their most prevalent conflicting bipartitions by calculating their gene support frequencies (GSF). GSF refers to the fraction of single-gene trees that recover a particular bipartition. Currently, RAxML can calculate GSF only for trees with full taxon representation. Since our data set contained partial gene trees, we conducted customs tests for determining GSF. To calculate GSF for NT (GSF_NT_) and AA (GSF_AA_) single-gene trees, we extracted subtrees for the taxa of interest in individual single-gene trees and counted the occurrence of various topologies. For example, consider that there are three taxa represented as A, B, and C, the reference rooted topology is “((A,B),C),” and the alternative rooted topology is “((A,C),B).” We counted how many single-gene trees supported “(A,B)” or “(A,C).” For reference and alternative topologies involving more than three taxa or sections, we conducted similar tests. For example, if the reference rooted topology is “(((A,B),C),D)” and the alternative rooted topology is “((A,B),(C,D)),” we counted how many single-gene phylogenies supported “((A,B),C)” as sister to D and how many single-gene phylogenies supported “(A,B)” and “(C,D)” as pairs of sister clades. For conflicting bipartitions at shallow depths in the phylogeny (i.e., among closely related species), we required all taxa to be present in a single-gene tree; for conflicting bipartitions near the base of the phylogeny (i.e., typically involving multiple sections), we required at least one species to be present from each section of interest. Scripts to determine GSF were written using functions provided in Newick Utilities, version 1.6 (142).

**(v) Filtering potential hidden paralogs.** Potential hidden paralogs among individual groups of orthologous genes can be identified by examining their ability to recover well-established monophyletic clades (45, 86, 87). To filter genes containing potential hidden paralogs among the 1,668 NT orthologs, we removed single genes that did not recover six well-established clades among *Aspergillus* and *Penicillium* species (47–49, 61). More specifically, we examined the 1,668 NT gene trees for monophyly of three *Aspergillus* clades (1, *Nigri*; 2, *Fumigati* and *Clavati*; and 3, *Aspergillus*) and three *Penicillium* clades (1, *Lanata-divaricata*; 2, *Chrysogena*; and 3, *Citrina*). We identified 337 NT gene trees that did not recover these six clades. Removal of these 337 NT genes resulted in a data matrix containing 1,331 NT genes. Using these 1,331 genes, we recalculated IC across the phylogeny and GSF at poorly supported bipartitions.

**(vi) Alternative trimming methods.** Alignment trimming methodologies can have a drastic effect on inferred phylogenies (88). To examine if our inferences were robust to different trimming methods, we also trimmed single-gene alignments using an entropy-based approach implemented in BMGE, version 1.12 (143). We used two different maximum entropy thresholds of 0.5 and 0.7, which we here refer to as BMGE_0.5_ and BMGE_0.7_, respectively. To examine the influence of this entropy-based alignment trimming approach, we used these additional data sets to reinfer species-level phylogenies using both the full 1,668-gene data matrix and the potential hidden paralog-filtered 1,331-gene data matrix.

**(vii) Topology tests.** To test the previously reported hypotheses of (a) the genus *Penicillium* being the sister group to *Aspergillus* section *Nidulantes* and (b) monophyly of narrow *Aspergillus* (sections *Nigri*, *Ochraceorosei*, *Flavi*, *Circumdati*, *Candidi*, and *Terrei*) (62), we conducted a series of tree topology tests using the 1,668-gene nucleotide data matrix using IQ-TREE (121). More specifically, we used the “GTR+F+I+G4” model and conducted the Shimodaira-Hasegawa (69) and the approximately unbiased (70) tests as specified with the “-au” parameter. These tests were conducted using 10,000 resamplings using the resampling estimated log-likelihood (RELL) method (144) as specified by the “-zb” parameter. We tested each hypothesis separately by generating the maximum likelihood topology under the constraint that the hypothesis is correct (specified using the “-z” parameter) and comparing its likelihood score to the score of the unconstrained maximum likelihood topology.

### Estimating divergence times.

To estimate the divergence times for the phylogeny of the Aspergillaceae, we analyzed our NT data matrix used the Bayesian method implemented in MCMCTree from the PAML package, version 4.9d (145). To do so, we conducted four analyses: we (i) identified genes evolving in a “clock-like” manner from the full data matrix, (ii) estimated the substitution rate across these genes, (iii) estimated the gradient and Hessian (146) at the maximum likelihood estimates of branch lengths, and (iv) estimated divergence times by Markov chain Monte Carlo (MCMC) analysis.

**(i) Identifying “clock-like” genes.** Currently, large phylogenomic data matrices that contain hundreds to thousands of genes and many dozens of taxa are intractable for Bayesian inference of divergence times; thus, we identified and used only those genes that appear to have evolved in a “clock-like” manner in the inference of divergence times. To identify genes evolving in a “clock-like” manner, we calculated the degree of violation of a molecular clock (DVMC) (71) for single-gene trees. DVMC is the standard deviation of root to tip distances in a phylogeny and is calculated using the following formula:

$$\text{DVMC}=\sqrt{\frac{1}{n-1}\overset{n}{\underset{i=1}{\sum }}{\left(t_{i}-\bar{t}\right)}^{2}}$$

where *t_i_* represents the distance between the root and species *i* across *n* species. Using this method, genes with low DVMC values evolve in a “clock-like” manner compared to those with higher values. We took the top-scoring 834 (50%) genes to estimate divergence times.

**(ii) Estimating substitution rate.** To estimate the substitution rate across the 834 genes, we used baseml from the PAML package, version 4.9d (145). We estimated substitution rate using a “GTR+G” model of substitutions (model = 7) and a strict clock model (clock = 1). Additionally, we point calibrated the root of the tree to 96 million years ago (mya) according to TimeTree (91), which is based on several previous estimates (50.0 mya [92], 96.1 mya [93], 146.1 mya [94]). We estimated a substitution rate of 0.04 substitutions per 10 million years.

**(iii) Estimation of the gradient and Hessian.** To save computing time, the likelihood of the alignment was approximated using a gradient and Hessian matrix. The gradient and Hessian refer to the first and second derivatives of the log-likelihood function at the maximum likelihood estimates of branch lengths (146) and collectively describe the curvature of the log-likelihood surface. Estimating gradient and Hessian requires an input tree with specified time constraints. For time constraints, we used the Aspergillus flavus-*Aspergillus oryzae* split (3.68 to 3.99 mya [94, 95]), the Aspergillus fumigatus-Aspergillus clavatus split (35 to 59 mya [94, 95]), the origin of the genus *Aspergillus* (43 to 85 mya [94, 96–99]), and the origin of Aspergillaceae (50 to 146 mya [92–94]) as obtained from TimeTree (91).

**(iv) Estimating divergence times using MCMC analysis.** To estimate divergence times using a relaxed molecular clock (clock = 2), we used the resulting gradient and Hessian results from the previous step for use in MCMC analysis using MCMCTree (145) and the topology inferred using the gene-partitioned approach and the 834-gene NT matrix from the top-scoring DVMC genes. To do so, a gamma distribution prior shape and scale must be specified. The gamma distribution shape and scale are determined from the substitution rate determined in step ii where shape is *a* = (*s*/*s*)^2^, scale is *b* = *s*/*s*^2^, and *s* is the substitution rate. Therefore, *a* = 1 and *b* = 25, and the “rgene_gamma” parameter was set to “1 25.” We also set the “sigma2_gamma” parameter to “1 4.5.” To minimize the effect of initial values on the posterior inference, we discarded the first 100,000 results. Thereafter, we sampled every 500 iterations until 10,000 samples were gathered. Altogether, we ran 5.1 million iterations [100,000 + (500 × 10,000)], which is 510 times greater than the recommended minimum for MCMC analysis (147). Last, we set the “finetune” parameter to 1.

### Statistical analysis and figure making.

All statistical analyses were conducted in R, version 3.3.2 (138). Spearman rank correlation analyses (148) were conducted using the “rcorr” function in the package Hmisc, version 4.1-1 (149). Stacked bar plots, bar plots, histograms, scatter plots, and box plots were made using ggplot2, version 2.2.1 (150). Intersection plots (also known as UpSet plots) were made using UpSetR, version 1.3.3 (151). The topological similarity heat map and hierarchical clustering were done using pheatmap, version 1.0.8 (152). Phylogenetic trees were visualized using FigTree, version 1.4.3 (153). The phylogenetic tree with the geological time scale was visualized using strap, version 1.4 (154). Artistic features of figures (e.g., font size, font style, etc.) were minimally edited using the graphic design software Affinity Designer (Serif, Nottingham, United Kingdom).

### Data availability.

All data matrices; species-level, single-gene phylogenies; and supplementary figures and files are available through the figshare repository https://doi.org/10.6084/m9.figshare.6465011. The genome sequence and raw reads of *Aspergillus spinulosporus* have been uploaded to GenBank as BioProject PRJNA481010.
